# Resident microbes of lactation rooms and daycares

**DOI:** 10.7717/peerj.8168

**Published:** 2019-12-13

**Authors:** Diana H. Taft, Samir Akre, Nicolas Madrid, Andre Knoesen, David A. Mills, Zachery T. Lewis

**Affiliations:** 1 Department of Food Science and Technology, University of California, Davis, Davis, CA, USA; 2 Department of Electrical and Computer Engineering, University of California, Davis, Davis, CA, USA; 3 Department of Viticulture and Enology, University of California, Davis, Davis, CA, USA

**Keywords:** Built environment microbiome, Lactation rooms, Daycares

## Abstract

Dedicated lactation rooms are a modern development as mothers return to work while still providing breastmilk to their absent infants. This study describes the built environment microbiome of lactation rooms and daycares, and explores the influence of temperature and humidity on the microbiome of lactation rooms. Sterile swabs were used to collect samples from five different sites in lactation rooms at University of California, Davis and from five different sites in daycares located in Davis, California. DNA from the swabs was extracted and the V4 region of the 16S rRNA gene was sequenced using Illumina MiSeq. Temperature and relative humidity data were collected on a subset of the lactation rooms. Sampled lactation rooms could be either dedicated lactation rooms or could also serve other functions (e.g., combined lactation room and restroom lounge). The majority of sequence reads were identified as belonging to family Moraxellaceae, with 73% of all reads included in analysis identified as an unknown species of *Acinetobacter*. Alpha diversity was analyzed using the Shannon index, while beta diversity was analyzed using unweighted and weighted UniFrac distance. The Jaccard distance was used to measure amount of change at sampling locations between time points for analysis of the impact of temperature and humidity on the microbiome. There were significant differences in the beta diversity of the microbiome of lactation rooms by room type. There were also significant differences in the beta diversity of the microbiome by sample collection location. There were no significant differences in either alpha or beta diversity associated with room temperature or humidity. Additional studies are needed to understand if the differences in lactation room type may result in differences in the breastmilk microbiome of milk collected in those rooms, and to what extent any such differences may influence the infant microbiome.

## Introduction

The rise of high-throughput sequencing has revolutionized the study of microbial communities, enabling new insight into the human microbiome and the built environment microbiome. The environment is a more important contributor to the human microbiome composition than host genetics (Rothschild et al., 2018). With more than half of the world population currently living in urban areas and the number of urban residents expected to reach two-thirds of the world population by 2050 (United Nations, 2018), the microbiome of the built environment is increasing in importance to human health. Compared to the outdoor environment, the built environment has limited microbial communities with a high percentage of human associated organisms (Gibbons, 2016). The shift from an outdoor environment to an indoor environment likely has important implications for human health, as shared housing explains more of the overlap in the microbiome composition of related individuals than shared genetic ancestry (Rothschild et al., 2018). Differences in the built environment microbiome can impact the human microbiome, and in turn influence human health. For example, differences in the indoor microbial environment correlate with differences in skin microbiome and risk of atopic sensitization in adolescents (Hanski et al., 2012). As a second example, childhood exposure to farming associated microbes, such as *Bacillus* and *Corynebacterium* species, is associated with reduced risk of allergy and asthma (Ege et al., 2011).

Because the infant microbiome is still developing, the influence of the built-environment microbiome may be greater during infancy than for older children and adults (Litvak & Bäumler, 2019). As such, it is important to understand the built-environment microbiota of infant related spaces. Two types of rooms with a potential to influence the developing infant microbiome are lactation rooms and daycares. Feeding infants expressed breastmilk is becoming an increasingly common practice (Binns et al., 2006), especially as mothers return to work after delivery. Pumped breastmilk microbiota are enriched in pathogens and depleted in *Bifidobacterium* compared to milk from mothers who have fed infants directly at the breast, although the reason or reasons for the microbiological differences remain unknown (Moossavi et al., 2019). One possible source of the microbes found in pumped milk is the lactation room itself, however, knowledge of the built-environment microbiome of lactation rooms is currently lacking. In the United States, women return to work after an average of 10 weeks maternity leave (Shepherd-Banigan & Bell, 2014), which necessitates pumping to continue to breastfeed exclusively for at least the recommended 6 months. For social and sanitary reasons, lactation rooms are ideally single purpose rooms for pumping. However, this is not practical at all work places and as a result lactation rooms may be co-located with restrooms or other purpose rooms. In addition, mothers’ return to work often means daycare for infants, introducing another environment from which infants may acquire microbes, including from one another. This work evaluates the built environment microbiome of daycares and lactation rooms, including different types of lactation rooms, and how temperature and humidity influences the microbiome of lactation rooms in effort to understand the microbes that infants may be exposed to either directly or indirectly.

## Materials and Methods

### Sample collection

Samples were collected from 28 University of California at Davis Lactation Rooms quarterly, excluding summers, between Spring 2015 and Fall 2016 and 4 daycares located in Davis, California in Winter 2016 and Fall 2016. The Spring 2015 quarter was from 26 March 2015 to 11 June 2015. The Fall 2015 quarter was from 21 September 2015 until 11 December 2015. The Winter 2016 quarter was from 4 January 2016 until 19 March 2016. The Spring 2016 quarter was from 24 March 2016 until 9 June 2016. The Fall 2016 quarter was from 19 September 2016 until 9 December 2016. Access to daycares for sampling was only available during the Winter and Fall 2016 quarters. All included rooms were within UC Davis property, so formal permission was not required. At the time, Shirley German managed the lactation rooms, Christine Pretti was the Director of Hutchison Child Development Center, Shelby Faria was the Director at La Rue Park, Janet Thompson was the Director of the Early Childhood Lab School at the Center for Child and Family Studies, and Tanya Chordas was the Director of the Russell Park Child Development Center. To collect samples, sterile swabs were first dampened with sterile phosphate buffered saline, then rubbed over the sampling location to collect any microbes present. Five samples per lactation room per sampling visit were collected from each lactation room; one each from a chair, a table, a pump, the floor, and the door to the room. Lactation rooms could be divided into categories based on other uses of the room, including sole purpose lactation rooms, a resident room, women’s restrooms, restroom lounges, single toilet unisex restrooms, and a shower room. There were five locations targeted for sample collection in daycares: a chair, a table, a toy, the floor, and the door to the room. After collection, swabs were placed in sterile 1.5 mL cyrovial tubes and stored at −80 °C until DNA extraction.

In addition, for a subset of ten lactation rooms, sensor nodes were placed near the door to enter the room to monitor room occupancy and select environmental conditions 24/7. Each custom-built sensor node (microcontroller, Cypress Semiconductor, PSoC5, CY8C5267AXI-LP051) contained a temperature and humidity sensor (Honeywell, HIH6130-021-001), a beam-break sensor (PIR Mini Sensor, Parallax 28033), a microSD card (Kingston 8 GB Class 4 MicroSDHC Card Flash Memory with SD Adapter SDC4/8 GB), and a battery (LiPo, 3.7v 2,500 mAh; Hunan Sounddon New Energy Co., Xiangtan City, China). The temperature and humidity data was collected every 15 min and stored on the microSD card. The beam-break sensor was used to determine when the room was in use by storing a time-stamp on the microSD card whenever the door was opened or closed. The microSD cards were retrieved from the sensor nodes to review the stored data. The sensor nodes were designed to be in an ultra-low-power state when measurements were not being taken to extend the battery life as long as possible, permitting measurements to be taken every 15 min for the entire time the sensors were in place. These sensor nodes were in place from early Fall 2015 to Fall 2016.

### DNA extraction and 16S rRNA gene sequencing

The base of the swabs were cut off using sterile scissors, and DNA was extracted using the Zymo Research Quick-DNA™ Fecal/Soil Microbe Miniprep Kit (Zymo Research, Irvine, CA, USA) according to the manufacturer’s instructions. For a negative control, seven swabs not used to collect samples were processed interspersed with the samples. An additional 24 kit control samples were extracted by completing all extraction steps without using a swab sample. Kit control samples were done using the same kit type and methods, but different lot numbers of reagents as kit controls were extracted, sequenced, and analyzed after the original sample extraction and sequencing were complete.

The barcoded F515 (5′-*NNNNNNNNGT*GTGCCAGC MGCCGCGGTAA-3′) and R806 (5′-GGACTACHVGGGTWTCTAAT-3′) primers were used to amplify the V4 region of the 16S ribosomal RNA gene, as previously described (Huda et al., 2014). After amplification, samples were purified using the Qiagen QIAquick PCR Purification Kit (Qiagen, Hilden, Germany) and pooled. The pooled library was then sequenced by the UC Davis Genome Center DNA Technologies Sequencing Core using 250-bp PE reads on the Illumina MiSeq DNA sequencing system (Illumina, San Diego, CA, USA). At least one negative control sample was included with every 95 samples.

The sequencing results were demultiplexed using Sabre (Joshi, 2011) and imported into QIIME2-2017.12 (Caporaso et al., 2010), which was used for trimming, denoising, taxonomic classification, and phylogenetic tree building. Bases before basepair 22 and after basepair 240 were trimmed from the forward read with bases before basepair 23 and after basepair 249 were trimmed from the reverse read. The trimmed reads were processed with DADA2 (Callahan et al., 2016). Taxonomy was assigned to representative sequences for each amplicon sequence variant (ASV) using the 99% Greengenes (McDonald et al., 2011) pre-generated naïve bayes classifier, and a phylogenetic tree was built using fasttree (Price, Dehal & Arkin, 2009) and midpoint rooted. The resulting ASV table, phylogenetic tree, representative sequences, and taxonomic assignments were exported from QIIME2 for additional analysis in R. These same steps were completed for the kit control samples, and any reads from ASVs found in both the samples and the kit controls were excluded from additional analysis.

### Statistical analysis

All statistical analysis was completed in R 3.4.3 statistical software (R Core Team, 2016). Samples with less than 1,000 reads were excluded from all analysis, as the rarefaction curve suggested that the number of species detected per sample reached a plateau for most samples at a read depth of approximately 1,000 (see Fig. S2). Prior to analysis, all samples were rarefied to a read depth of 1,040 reads, as this was the read depth of the sample with the fewest reads after excluding samples with <1,000 reads. For alpha and beta diversity analyses, samples were stratified by quarter (Spring 2015, Fall 2015, Winter 2016, Spring 2016 and Fall 2016). Only the earliest successfully sequenced sample from each sampling site (e.g., sample collected at a specific location in a room) in each quarter was included in the analysis. The vegan 2.5.1 package (Oksanen et al., 2017) was used to calculate the Shannon index. Kruskal–Wallis test was used to test for differences in alpha diversity by room type and collection location, followed by Dunn’s test if the Kruskal–Wallis test was significant to determine which room types and collection locations differed from each other. A two-tailed *p* < 0.05 was considered significant for the Kruskal–Wallis test and a Bonferroni adjusted two-tailed *p* < 0.05 was considered significant for Dunn’s test. For beta-diversity analysis, the weighted and unweighted UniFrac distance between samples was calculated using the GUniFrac 1.1 package (Chen et al., 2012). The vegan package implementation of non-metric multidimensional scaling (NMDS) was used to generate ordination plots, if stress was less than 0.2, a 2 dimensional plot was used. Otherwise the number of dimensions was increased to obtain a plot that was a reasonable representation of the data. Beta dispersion was calculated using the betadisper function in the vegan package (Dixon, 2003). ANOVA was used to test for differences in beta dispersion by room type and by sample collection location. If there were no significant differences in beta dispersion, PERMANOVA as implemented by adonis was run to test for statistical significance in beta diversity by room type and sample collection location, with the interaction term included in the model. As the study design was unbalanced for all quarters, PERMANOVA was not run if the beta dispersion differed significantly by room type or sample collection location if the group with smaller sample size had greater dispersion as this will tend to make PERMANOVA too liberal (Anderson & Walsh, 2013).

To explore the relationship between temperature and humidity and the built environment microbiome, the collection day and room of all successfully sequenced samples was compared to the days and rooms with temperature and humidity sensor. For each sample collected while a sensor was present in the room, the average temperature and humidity for the day of sample collection was calculated. To test for an association between alpha diversity and temperature and humidity, linear mixed effects models were run using the lme command in the nlme 3.1-137 R package (Pinheiro et al., 2014) with the Shannon index as the outcome variable and the average room temperature and the average room humidity as fixed variables. Room was included as a random variable. For beta diversity analysis, first the same set of first samples was used in an NMDS based on the weighted and unweighted UniFrac distance measures. Then the association of the ordination with the continuous average temperature and continuous average humidity were tested in separate ordisurf models (as implemented in the vegan package).

Finally, we sought to understand if greater changes in temperature or humidity were associated with greater changes with the built environment microbiome. For any room/location combinations that had multiple samples successfully sequenced with available sensor data, the Jaccard distance between the samples between the repeated measurements. Then the absolute value of the difference between the average temperature and the average humidity at the two time points was calculated (magnitude of change in temperature and humidity). The association between Jaccard distance and magnitude of change in temperature and humidity was calculated using a linear mixed effects model (lme command) with room included as random effect to account for multiple sampling locations within the same room.

## Results

### Samples

There were a total of 460 samples collected from lactation rooms and daycares for this study, seven negative controls, and 24 extraction kit controls. The kit controls had 11 different ASVs present, five of which were present in the other samples. These five ASVs were identified as *Escherichia coli*, an unknown species of *Pseudomonas*, *Pseudomonas fragi, Pseudomonas veronii*, and an unknown species of Lactococcus. These ASVs represented 1.1% of all reads from samples included in the final analysis, ranging from 0% to 6.7% of reads on a per sample basis, with a median of 1% of reads removed from each sample included in additional analysis. One negative control had an unexpectedly high number (>42,000 reads) of reads after sequencing, suggesting contamination in the set of samples either extracted or amplified at the same time as the negative control, as a result all samples handled in a batch with the contaminated negative control were excluded from additional analysis, leaving 388 samples. Of these samples, 230 had a read depth greater than 1,000 reads. Excluding samples with less than 1,000 reads removed all additional negative controls, as after excluding the likely contaminated samples, the negative control with the next most reads had only 48 reads. Of the samples excluded due to low read depth, half had a read depth at or below that observed in the negative controls. An additional 17 samples were excluded from the analysis stratified by time because sampling was completed twice in the same rooms at the same collection locations in some quarters, leaving 213 samples for inclusion in the diversity by quarter analyses. A total of 55 samples were collected from rooms with sensor data available on the day of collection, 39 of these were collected from unique room and collection locations and were included in the alpha and beta diversity analyses. An additional 16 samples were collected from the same room and location as an earlier sample, resulting in 32 samples available for the change in temperature and humidity analysis, and a total of 221 samples included in one or both analyses. Table 1 summarizes the samples included in each analysis. Demultiplexed sequencing data of all samples included in the analysis are available at SRA database with BioProject accession number PRJNA547638. Table 2 summarizes the results by quarter discussed below.

**Table 1:** Sample number by room, location type, and quarter. Number of samples included in analyses, by room type and sample collection location. Each quarter had a separate analysis completed, TH stands for temperature humidity. All rooms were either official UC Davis Lactation Rooms or Daycares, but some Lactation Rooms also had additional functions.

Room type	Spring 2015	Fall 2015	Winter 2016	Spring 2016	Fall 2016	TH diversity	TH change
	*n* = 79	*n* = 42	*n* = 48	*n* = 11	*n* = 33	*n* = 39	*n* = 32 (16 pairs)
Dedicated lactation room (chair/door/floor/pump/table)	11/9/1/9/7	6/4/7/6/6	5/4/4/7/4	0/1/0/1/0	2/2/2/5/2	5/2/5/4/3	6/2/4/2/0
Resident room (chair/door/floor/pump/table)	1/1/1/1/1	1/1/1/1/1	NA	NA	1/0/1/1/1	1/1/1/1/1	2/2/0/2/2
Women’s restroom (chair/door/floor/pump/table)	1/3/1/2/1	0/0/1/0/1	NA	0/0/1/0/1	NA	NA	NA
Restroom lounge (chair/door/floor/pump/table)	5/4/3/3/3	1/0/0/0/1	1/1/1/2/1	1/0/1/0/0	1/2/1/2/2	2/2/1/3/3	2/2/2/2/2
Unisex restroom (chair/door/floor/pump/table)	2/1/2/1/2	1/1/0/1/1	2/2/2/1/0	1/1/1/0/0	NA	1/1/0/1/1	NA
Shower room (chair/door/floor/pump/table)	0/0/1/1/1	NA	0/1/1/1/0	0/0/1/0/1	NA	NA	NA
Daycare (chair/door/floor/toy/table)	NA	NA	1/2/1/2/2	NA	1/3/2/0/2	NA	NA

**Table 2:** Summary of alpha and beta-diversity results by quarter.

Quarter	Median Shannon index values by room type	*p*-Value	Median Shannon index values by collection location	*p*-Value	Adonis *R*^2^ values, unweighted UniFrac	*p*-Values	Adonis *R*^2^ values, weighted UniFrac	*p*-Values
Spring 2015	Lactation room - 0.63 Resident room - 0.63 Restroom - 0.78 Restroom lounge - 0.77 Restroom, unisex - 1.1 Shower room - 0.99	0.091	Chair - 0.72 Door - 0.61 Floor - 0.87 Pump - 0.66 Table - 0.69	0.075	Room type - 0.085 Collection location - 0.071	0.059 0.043	N/A	N/A
Fall 2015	Lactation room - 0.62 Resident room - 0.57 Restroom - 1.1 Restroom lounge - 3.3 Restroom, unisex - 0.68	0.11	Chair - 0.64 Door - 0.62 Floor - 1.4 Pump - 0.62 Table - 0.56	0.14	Room type - 0.13 Collection location - 0.18	0.036 0.002	Room type - 0.59 Collection location - 0.065	0.001 0.11
Winter 2016	Daycare - 2.4 Lactation room - 0.66 Restroom lounge - 1.4 Restroom, unisex - 0.61 Shower room - 0.99	0.0068	Chair - 0.67 Door - 0.77 Floor - 0.99 Pump - 0.89 Table - 0.65 Toy - 1.6	0.90	Room type - 0.16 Collection location - 0.093	0.002 0.55	Room type - N/A Collection location - 0.11	N/A 0.32
Fall 2016	Daycare - 1.0 Lactation room - 0.73 Resident room - 0.73 Restroom lounge - 0.73	0.83	Chair - 0.73 Door - 0.60 Floor - 1.5 Pump - 0.76 Table - 0.70	0.19	Room type - 0.14 Collection location - 0.18	0.009 0.006	Room type - 0.039 Collection location - 0.21	0.042 0.969

### Spring 2015

**Figure 1:**
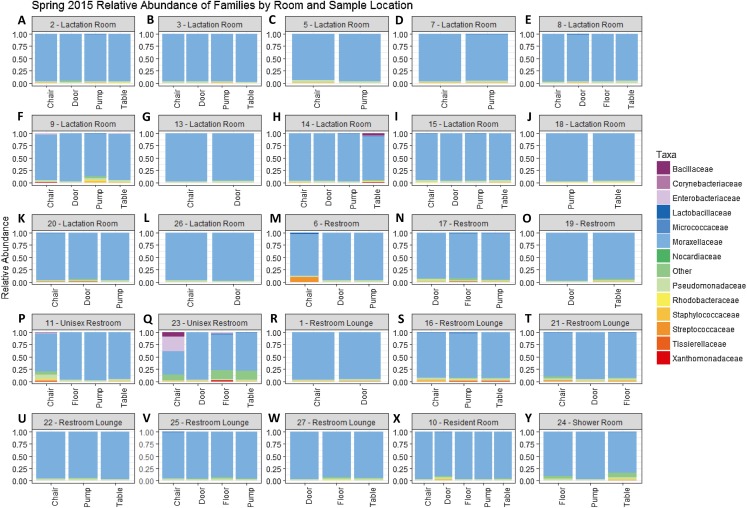
Family level relative abundance of taxa by room and sampling location in Spring 2015. All rooms were used a lactation rooms, dedicated lactation rooms are captioned “Lactation Room.” (A–L) Samples from dedicated lactation rooms, grouped by room. (M–O) Samples from lactation rooms co-located with women’s restrooms. (P–Q) Samples from lactation rooms co-located with unisex restrooms. (R–W) Samples from lactation rooms co-located with women’s restroom lounges. (X) Samples from lactation room co-located with a resident room. (Y) Samples from a lactation room co-located with a shower room.

**Figure 2:**
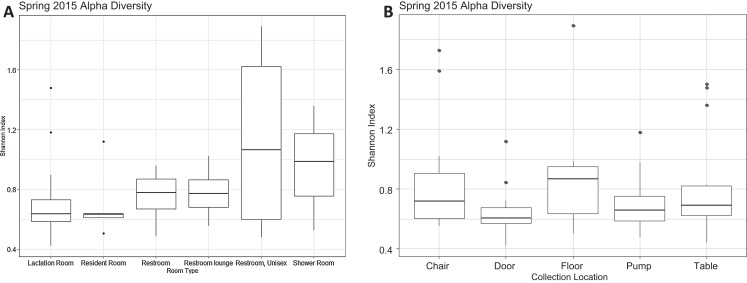
Alpha diversity Spring 2015. (A) Alpha diversity as measured by Shannon’s Index did not differ significantly by room type (Kruskal–Wallis, *p* = 0.091). All rooms were used as lactation rooms, the label “Lactation Room” refers to dedicated lactation rooms. (B) Alpha diversity as measured by Shannon index did not differ significantly by collection location (Kruskal–Wallis, *p* = 0.075).

There were a total of 79 samples successfully sequenced from 25 lactation rooms in Spring 2015. The majority of reads in most samples, regardless of room type or sample collection location, belonged to family Moraxellaceae (Fig. 1). A single sample, the chair sample from room 23, had markedly higher levels of Enterobacteriaceae than all other samples at this time point. There was no significant association between alpha diversity as measured by the Shannon index and sample collection location (Kruskal–Wallis, *p* = 0.075), or between Shannon index values and room type (Kruskal–Wallis, *p* = 0.091) (Fig. 2).

**Figure 3:**
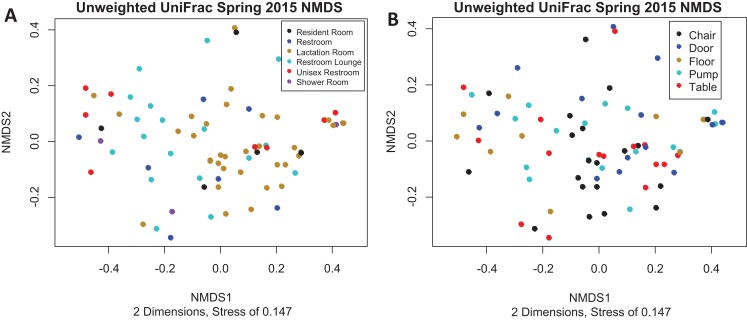
Unweighted UniFrac NMDS Spring 2015. (A) Unweighted UniFrac NMDS with points colored by room type. All rooms were used as lactation rooms, the label “Lactation Room” refers to dedicated lactation rooms. (B) Unweighted UniFrac NMDS with points colored by sample collection location.

There is no visible separation of points on the NMDS plot based on unweighted UniFrac for the Spring 2015 samples by either room type or sample collection location (Fig. 3). There were no significant differences in beta-dispersion by room type (*p* = 0.59) or sample collection location (*p* = 0.87). There were significant differences in unweighted UniFrac beta diversity by sample collection location (PERMANOVA, *p* = 0.043) but not by room type (PERMANOVA, *p* = 0.059). The room 23 chair sample with high levels of Enterobacteriaceae skewed the weighted UniFrac analysis, with all other points appearing on top of each other in the weighted UniFrac NMDS, therefore, the weighted UniFrac results were not analyzed further. Because remaining quarters generally showed a similar pattern, figures from other quarters can be found in Supplemental Information.

### Fall 2015

There were a total of 42 samples successfully sequenced from 13 lactation rooms in Fall 2015. As with Spring 2015, most samples were dominated with reads mapping to Moraxellaceae, however, there were some samples with relatively low levels of Moraxellaceae and more samples with substantial levels of other families present (Fig. S3). There was no significance association between alpha diversity as measured by Shannon index and room type (Kruskal–Wallis, *p* = 0.11) or sample collection location (Kruskal–Wallis, *p* = 0.14) (Fig. S4).

There was no visible separation of points on NMDS plot based on unweighted UniFrac for the Fall 2015 samples by room type, however, there did appear to be some separation between the floor samples and the samples collected at other locations (5). There was no significant differences in beta-dispersion by collection location (*p* = 0.79), but there was a significant difference by room type (*p* = 0.0072). As the room type with the larger sample size had greater dispersion, PERMANOVA will tend to be too conservative (Anderson & Walsh, 2013) so we proceeded with the PERMANOVA despite the differences in dispersion. As in Spring 2015, there were significant difference in beta diversity as measured by unweighted UniFrac by collection location (*p* = 0.002), but now there was also a significant difference by room type (PERMANOVA, *p* = 0.036) as well. Unlike the unweighted UniFrac ordination, the weighted UniFrac ordination did not appear to have any separation of points by room type or collection location (Fig. S6). There were no significant differences in beta-dispersion by room type (*p* = 0.10) or by collection location (*p* = 0.46). Despite the lack of visual separation on the NMDS, there was a significant difference in beta diversity by room type (PERMANOVA, *p* = 0.001). However, there was not a significant difference by sample collection location (*p* = 0.11).

### Winter 2016

There were a total of 40 samples collected and successfully sequenced from 14 lactation rooms and eight samples collected and successfully sequenced from two daycares in Winter 2016, for a total of 48 samples. As with previous quarters, Moraxellaceae was the dominant taxa in most lactation room samples. Three of the 8 day care samples were also dominated by Moraxellaceae, but the remaining samples appeared more diverse (Fig. S7). Consistent with this result, there was a significant difference in alpha diversity as measured by the Shannon Index by room type (Kruskal–Wallis, *p* = 0.0068) (Fig. S8). Post-hoc comparison confirmed that the significant differences were between daycare samples and dedicated lactation room samples (Dunn’s test, *p* = 0.0037) and between daycare samples and unisex restroom samples (*p* = 0.013). There was no significant difference in Shannon’s index by sample collection location (Kruskal–Wallis, *p* = 0.90) (Fig. S8).

Daycare samples tended to visually separate from samples from other room types on the unweighted UniFrac NMDS (Fig. S9). There were no significant differences in beta-dispersion by room type (*p* = 0.50) or sample collection location (*p* = 0.18). However, unlike in previous quarters, there was no significant difference in unweighted UniFrac beta diversity by sample collection location (PERMANOVA, *p* = 0.55), although there was a significant difference by room type (*p* = 0.002). As with the unweighted UniFrac, the weighted UniFrac NMDS showed some visual separation of the daycare samples from the other room types. There was no visible separation in weighted UniFrac distance by sample collection location (Fig. S10). While there was no significant difference in beta-dispersion by sample collection location (*p* = 0.46) for the weighted UniFrac analysis, there was a significant difference by room type (*p* = 0.0078). Furthermore, the room types with the fewest samples had large dispersion and as a result, PERMANOVA testing for differences has the potential to be too liberal (Anderson & Walsh, 2013), and so room type was not included in the weighted UniFrac PERMANOVA model. There was no significant association between beta diversity as measured by weighted UniFrac and sample collection location (PERMANOVA, *p* = 0.325).

### Spring 2016

There were only 11 total samples from 6 lactation rooms successfully sequenced from Spring 2016. As with other quarters, the majority of the samples were dominated by Moraxellaceae (Fig. S11). Because most room types and most sample collection locations had fewer than 3 samples per category, additional statistical analysis was not completed for this quarter.

### Fall 2016

There were 26 samples from 10 lactation rooms and 8 samples from 4 daycares that were successfully sequenced in Fall 2016. Moraxellaceae remained the dominant taxa in most lactation rooms, and unlike in Winter 2016, Moraxellaceae was now the dominant taxa in all daycare samples (Fig. S12). Because there was only a single sample from a single stall women’s restroom collected in this quarter, this sample was excluded from additional analysis. There were no significant differences in alpha diversity as measured by the Shannon index by room type (Kruskal–Wallis, *p* = 0.83) or by sample collection location (*p* = 0.19) (Fig. S13).

Despite the more comparable levels of Moraxellaceae between daycares and lactation rooms, the daycares did appear somewhat visually separate from the lactation rooms on the unweighted UniFrac NMDS. There also seemed to be some separation by sample collection location, particularly between pump and floor samples (Fig. S14). There were no significant differences in beta-dispersion by collection location (*p* = 0.98) but there were significant differences by room type (*p* = 0.0078), but the room types with more samples had larger dispersion making PERMANOVA conservative. There were significant differences in beta diversity as measured by unweighted UniFrac by both room type (PERMANOVA, *p* = 0.009) and by sample collection location (*p* = 0.006). Using weighted UniFrac distance, there was no clear separation by either room type, but the pump collection location did appear to be different from other collection locations (Fig. S15). There was no significant difference in beta-dispersion of weighted UniFrac distance by room type (*p* = 0.48), however, there was a significant difference in beta-dispersion by sample collection location (*p* = 0.027) but the location with the greatest dispersion also had the most samples, tending to make PERMANOVA conservative. There was no significant difference in beta diversity based on weighted UniFrac distance by room type (PERMANOVA, *p* = 0.97), but there was a significant difference by collection location (PERMANOVA, *p* = 0.042).

### Moraxellaceae genera and species present

Because Moraxellaceae was dominant in many sampling locations and time points, further description of the ASVs identified as belonging to this family is warranted. Of all the reads in the rarefied ASV table, 84% were identified as belonging to family Moraxellaceae. The majority of these reads were found in two ASVs. The first ASV was an unknown species of *Acinetobacter* found in 97% of all samples, and represented 73% of all reads in the rarefied ASV table and 86% of all Moraxellaceae reads. The second ASV was identified as *A. johnsonii* found in 93% of all samples, and represented 9% of all reads in the rarefied ASV table and 11% of all Moraxellaceae reads. The remaining ASVs were primarily identified as *Acinetobacter*, but a few ASVs were identified as belonging to other genera (Table 3).

**Table 3:** The genus and species of all ASVs identified as family Moraxellaceae. There were 125 ASVs identified as belonging to family Moraxellaceae in the rarefied ASV table. The majority of reads mapped to two ASVs, one an unknown species of *Acinetobacter* and one identified as *A. johnsonii*.

Family Moraxellaceae species	Number of ASVs
Unknown genus and species	2
*Acinetobacter*, unknown species	25
*Acinetobacter guillouiae*	76
*Acinetobacter johnsonii*	5
*Acinetobacter lwoffi*	3
*Acinetobacter rhizosphaerae*	3
*Acinetobacter schindleri*	1
*Alkanindiges*, unknown species	1
*Enhydrobacter*, unknown species	2
*Moraxella*, unknown species	1
*Perlucidibaca*, unknown species	2
*Psychrobacter*, unknown species	2
*Psychrobacter pulmonis*	2

### Alpha and beta diversity association with temperature and humidity

The mean average temperature of lactation rooms on the day of sample collection was 20.9 °C (range 18.3–22.4°C) for the samples included in this analysis. The mean average humidity of lactation rooms on the day of sample collection was 35.6% (range 21.2–68.3%) for the samples included in this analysis. There was no significant association between Shannon index values and temperature (*p* = 0.22) or humidity (*p* = 0.77).

The unweighted UniFrac NMDS ordination had two dimensions and a stress of 0.0962. There was no significant association between the ordination results and the average room temperature on day of collection (*p* = 0.16) or the average room humidity on day of collection (*p* = 0.41) by ordisurf (non-significant, resulting figure not shown). The weighted UniFrac NMDS ordination had two dimensions and a stress of 0.0413. There was no significant association between the ordination results and temperature (*p* = 0.109) or humidity (*p* = 0.75) by ordisurf (non-significant, resulting figure not shown).

### Community change association with temperature and humidity

A Jaccard distance of 0 indicates identical samples and a Jaccard distance of 1 indicates no overlap of replicon sequence variants between repeated samples from the same location. The Jaccard distance between repeated samples varied widely, with a median value of 0.232 (range 0.040–0.99). There was no significant association between the Jaccard distance and the magnitude of humidity change (lme value = 0.0206, *p* = 0.083) or between the Jaccard distance and the magnitude of the temperature change (lme value = −0.124, *p* = 0.51).

## Discussion

The dominant taxon from most samples in lactation rooms and daycares was family Moraxellaceae, a member of phylum Proteobacteria. Members of Moraxellaceae are widespread in nature, with possible habitats including soil, water, food, and the skin and mucous members of animals, including humans (Teixeira & Merquior, 2014). While most species in this taxa are harmless to humans, a few may cause illness in humans (Teixeira & Merquior, 2014; Visca, Seifert & Towner, 2011). As a significant minority of bacteria in the office built environment derives from human skin bacteria even without direct contact between humans and the sampled surface (Chase et al., 2016), and the microbiomes of public restroom surfaces normally touched by skin found that the human skin microbiome was the major contributor to the built environment microbiome (Flores et al., 2011), we speculate that the Moraxellaceae found in lactation rooms and daycares may arise from human skin, especially since the sampling locations in this study all involved surfaces with direct human contact. This is further supported by the fact that the majority of Moraxellaceae detected in this study belonged to genus *Acinetobacter*, particularly *A. johnsonii. Acinetobacter* are normal members of the skin microflora, and *A. johnsonii* is one of the most frequently isolated species (Seifert et al., 1997). In more recent studies of the built environment, *Acinetobacter* are commonly found in kitchens and are also thought to be associated with outdoor environments (Adams et al., 2015). Additional studies examining both the skin microbiome of lactation room users and daycare attendees as well as built environment microbial sampling are needed to confirm this hypothesis.

In addition to near-ubiquitous domination by Moraxellaceae, high relative amounts of other taxa relevant to human health were observed in individual samples, including samples with higher levels of Lactobacillaceae, Enterobacteriaceae, Staphylococcaceae, and Streptococcaceae. While there were no clear explanatory patterns behind the samples containing these other taxa, and while this study provides no information on the viability of these organisms, this does raise the possibility of transfer of these organisms to individuals via the built environment. Further studies investigating the viability and strain-level identity of the organisms are needed to help judge the risks or benefits exposure to these organisms may create to both the individuals using these spaces and their infants.

We observed differences in beta diversity as measured with unweighted UniFrac distance by room type for all quarters with sufficient samples for this analysis except Spring 2015. This relationship was also seen for the two quarters (Fall 2015 and Fall 2016) where the weighted UniFrac by room type analysis could be completed. The differences in the built environment microbiome by room type may represent differences in the microbiome of the people who use the room, as recent work has demonstrated humans have unique microbial clouds that settle in the built environment (Meadow et al., 2015). This suggests that future studies should examine the microbiome of the full range of individuals who may access lactation rooms, and not just nursing mothers. As with room type, sample collection location was also associated with unweighted UniFrac distances in most quarters, but was never associated with weighted UniFrac distance. As work in office buildings has demonstrated that type of surface has little influence on built environment microbiome compared to sample location (Chase et al., 2016), these differences are more likely due to differences in how people interact with the different sampling location. Therefore, work focused on sampling the pumps in lactation rooms may be more relevant to the development of the infant gut microbiome.

A limitation of this study is the number of samples that failed sequencing or were contaminated. This resulted in a design unbalanced by sample location, and made it more difficult to adjust for the repeated measure sampling caused by collecting five samples per room. As an example, the beta diversity analysis did not use room as a strata grouping variable because of the frequency of rooms with only one or two successfully sequenced sample. As specifying strata restricts permutations by strata level, specifying strata as room would curtail the possible permutations when the majority of samples in a given room for a given quarter failed sequencing. However, repeated measures within a room were accounted for during the analysis comparing the Jaccard distance between repeated samples at the same site to the magnitude of temperature and humidity changes. There was not a significant relationship between either temperature or humidity and alpha diversity, beta diversity, or change in temperature or humidity in lactation rooms, similar to what was observed in the office environment (Chase et al., 2016). Chase et al. (2016) speculate that the reason they did not observe any association between temperature or humidity and the built environment microbiome was the narrow range over which temperature and humidity fluctuated in their samples. As the rooms included in this study also had a narrow range of temperatures but a larger range of relative humidity, this may be why while there was no association with temperature, the relationship between change in humidity and change in the microbiome neared significance. This is inconsistent with other studies which have reported associations between humidity and the built environment microbiome (Kembel et al., 2012; Leung et al., 2014). Another reason for the difference between our work and prior work regarding humidity is that lactation rooms tended to be drier, Leung et al. (2014) included outdoor samples with higher relative humidity and the Hong Kong subway system studied by Kembel et al. (2012) had a mean relative humidity close to the upper limit of relative humidity observed in this study. Unlike the studies where relative humidity was associated with the built-environment microbiome, this study had a mean relative humidity below the level at which bacteria can grow (De Goffau et al., 2009).

## Conclusion

The microbiome of lactation rooms varies with the room type, for example, dedicated lactation rooms have differences in their built environment microbiome compared to lactation rooms that also fulfill other purposes. In addition, the built environment microbiome of lactation rooms can differ from that of daycares. The built environment microbiome of lactation rooms is not constant over time, but these differences were not significantly associated with changes in temperature or humidity. More work is needed to understand if differences in lactation room microbiomes results in differences of the breastmilk microbiome fed to infants, if lactation rooms that also serve other purposes have differing influences on the breastmilk microbiome, and what the potential risks and benefits of exposure to these built-environment microbes might be.

## Supplemental Information

10.7717/peerj.8168/supp-1 Supplemental Information 1 Picture of two of the sensors placed in lactation rooms. Photo credit: Diana H. Taft.

10.7717/peerj.8168/supp-2 Supplemental Information 2 Rarefaction curve of sampling depth. All samples have adequate sampling depth to assess diversity at 5,000 reads, most samples have adequate sequencing depth at 1,000 reads. Therefore, a rarefaction depth of 1,040 reads was chosen to retain as many samples as possible while still having adequate sampling depth for most samples.

10.7717/peerj.8168/supp-3 Supplemental Information 3 Family level relative abundance of taxa by room and sampling location in Fall 2015. All rooms were used a lactation rooms, dedicated lactation rooms are captioned “Lactation Room.”

10.7717/peerj.8168/supp-4 Supplemental Information 4 Alpha Diversity Fall 2015. (A) Alpha diversity as measured by Shannon index by room type (*p* = 0.112). All rooms were used as lactation rooms, the label “Lactation Room” refers to dedicated lactation rooms. (B) Alpha diversity as measured by Shannon Index by sample collection location (*p* = 0.141).

10.7717/peerj.8168/supp-5 Supplemental Information 5 Unweighted UniFrac NMDS Fall 2015. (A) Unweighted UniFrac NMDS with points colored by room type. All rooms were used as lactation rooms, the label “Lactation Room” refers to dedicated lactation rooms. (B) Unweighted UniFrac NMDS with points colored by sample collection location.

10.7717/peerj.8168/supp-6 Supplemental Information 6 Weighted UniFrac NMDS Fall 2015. (A) Weighted UniFrac NMDS with points colored by room type. All rooms were used as lactation rooms, the label “Lactation Room” refers to dedicated lactation rooms. (B) Weighted UniFrac NMDS with points colored by sample collection location.

10.7717/peerj.8168/supp-7 Supplemental Information 7 Family level relative abundance of taxa by room and sampling location in Winter 2016. All rooms were daycares or used as lactation rooms, dedicated lactation rooms are captioned “Lactation Room.”

10.7717/peerj.8168/supp-8 Supplemental Information 8 Alpha Diversity Winter 2016. (A) Alpha diversity as measured by Shannon index by room type (Kruskal–Wallis, *p* = 0.0068, Dunn’s test indicated a difference was between daycares and lactation rooms (*p* = 0.0037) and between daycares and unisex restrooms (*p* = 0.013). All rooms were daycares or used as lactation rooms, the label “Lactation Room” refers to dedicated lactation rooms. (B) Alpha diversity as measured by Shannon Index by sample collection location (*p* = 0.90).

10.7717/peerj.8168/supp-9 Supplemental Information 9 Unweighted UniFrac NMDS Winter 2016. (A) Unweighted UniFrac NMDS with points colored by room type. All rooms were daycares or used as lactation rooms, the label “Lactation Room” refers to dedicated lactation rooms. (B) Unweighted UniFrac NMDS with points colored by sample collection location.

10.7717/peerj.8168/supp-10 Supplemental Information 10 Weighted UniFrac NMDS Winter 2016. (A) Weighted UniFrac NMDS with points colored by room type. All rooms were daycares or used as lactation rooms, the label “Lactation Room” refers to dedicated lactation rooms. (B) Weighted UniFrac NMDS with points colored by sample collection location (PERMANOVA, *p* = 0.175).

10.7717/peerj.8168/supp-11 Supplemental Information 11 Family level relative abundance of taxa by room and sampling location in Spring 2016. All rooms were used as lactation rooms, dedicated lactation rooms are captioned “Lactation Room.”

10.7717/peerj.8168/supp-12 Supplemental Information 12 Family level relative abundance of taxa by room and sampling location in Fall 2016. All rooms were daycares or used as lactation rooms, dedicated lactation rooms are captioned “Lactation Room.”

10.7717/peerj.8168/supp-13 Supplemental Information 13 Alpha Diversity Fall 2016. (A) Alpha diversity as measured by Shannon index by room type (*p* = 0.83). All rooms were daycares or used as lactation rooms, the label “Lactation Room” refers to dedicated lactation rooms. (B) Alpha diversity as measured by Shannon Index by sample collection location (*p* = 0.19).

10.7717/peerj.8168/supp-14 Supplemental Information 14 Unweighted UniFrac NMDS Fall 2016. (A) Unweighted UniFrac NMDS with points colored by room type. All rooms were daycares or used as lactation rooms, the label “Lactation Room” refers to dedicated lactation rooms. (B) Unweighted UniFrac NMDS with points colored by sample collection location.

10.7717/peerj.8168/supp-15 Supplemental Information 15 Weighted UniFrac NMDS Fall 2016. (A) Weighted UniFrac NMDS with points colored by room type. All rooms were daycares or used as lactation rooms, the label “Lactation Room” refers to dedicated lactation rooms. (B) Weighted UniFrac NMDS with points colored by sample collection location. Because of significant differences in betadispersion by sample collection location, sample collection location was not included in the PERMANOVA model.

## Additional Information and Declarations

### Field Study Permissions

The following information was supplied relating to field study approvals (i.e., approving body and any reference numbers): Christine Pretti (Director) approved sample collection at the Hutchison Child Development Center, Shelby Faria (Director) approved sample collection at La Rue Park, Janet Thompson (Director) approved sample collection at the Early Childhood Lab School at the Center for Child & Family Studies, and Tanya Chordas (Director) approved sample collection at Russell Park Child Development Center.

### Data Availability

The following information was supplied regarding data availability: Demultiplexed sequencing data of all samples included in the analysis are available at the SRA database with BioProject accession number PRJNA547638.
