# Supplementary figures and images for: Resident microbes of lactation rooms and daycares
Source: PeerJ. 2019 Dec 13;7:e8168. doi: 10.7717/peerj.8168 (PMC6913265; doi:10.7717/peerj.8168)

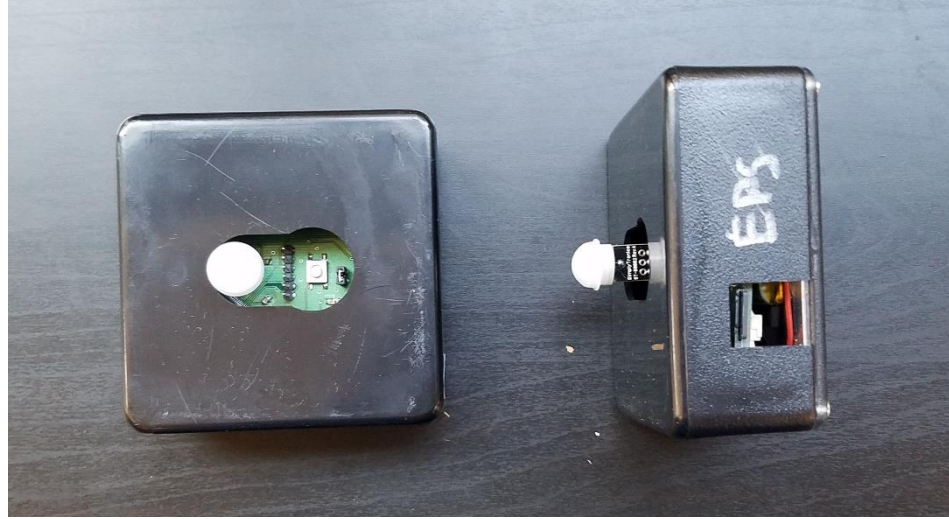

Supplement: Supplemental Information 1 — Photo credit: Diana H. Taft. [file peerj-07-8168-s001.pdf]

**Rarefaction Curve**

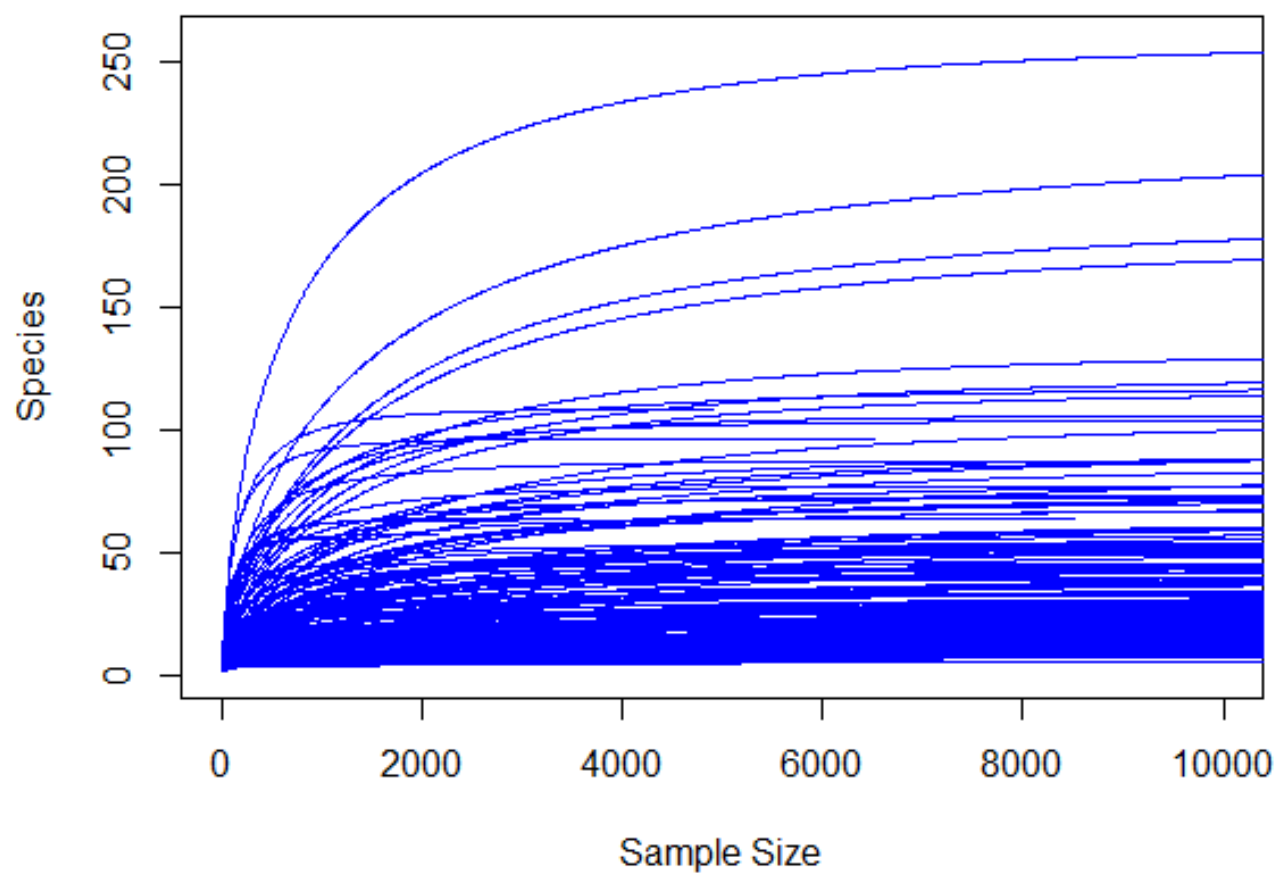

Supplement: Supplemental Information 2 — All samples have adequate sampling depth to assess diversity at 5,000 reads, most samples have adequate sequencing depth at 1,000 reads. Therefore, a rarefaction depth of 1,040 reads was chosen to retain as many samples as possible while still having adequate sampling depth for most samples. [file peerj-07-8168-s002.pdf]

Fall 2015 Relative Abundance of Families by Room and Sample Location

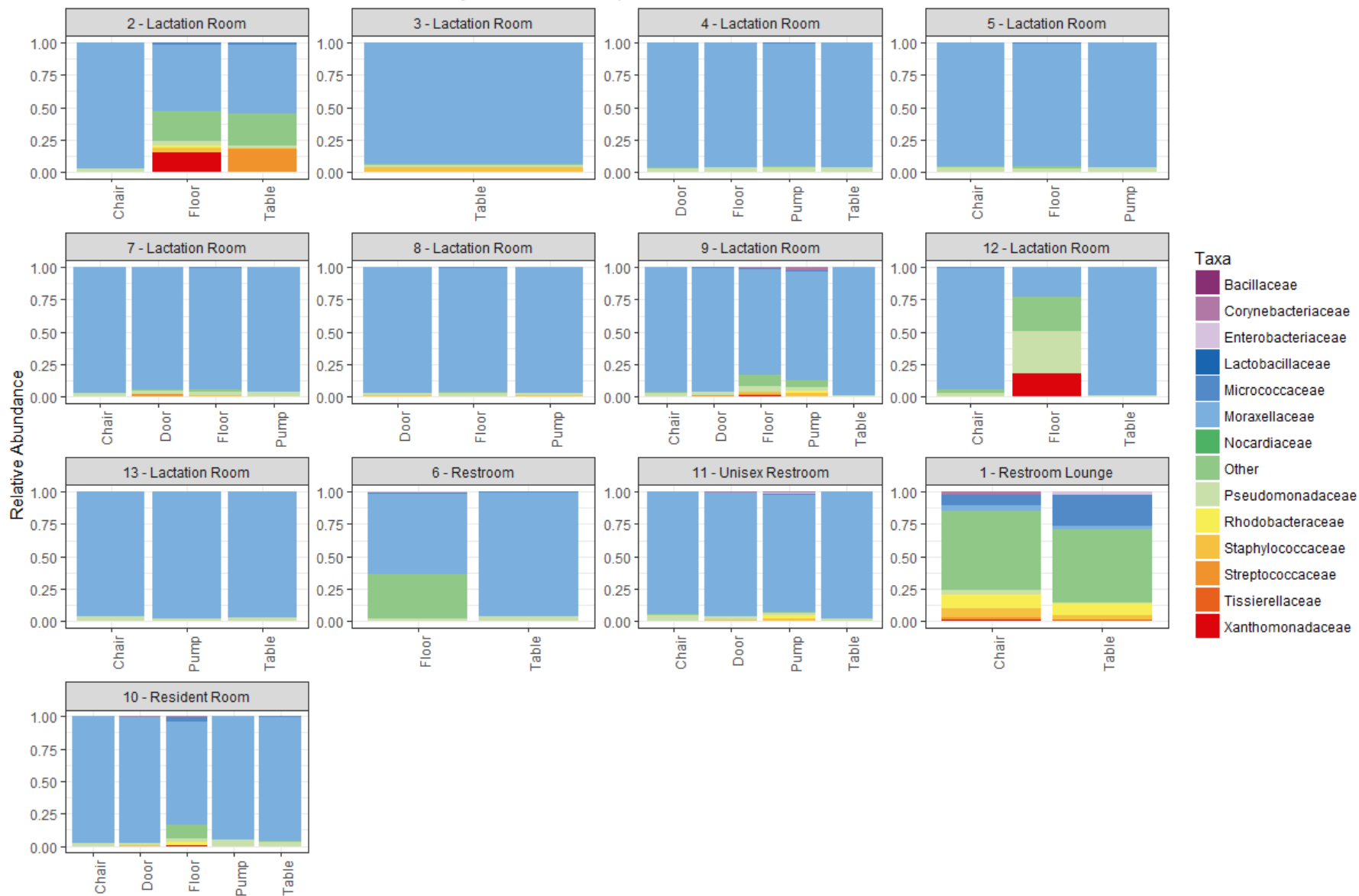

Supplement: Supplemental Information 3 — All rooms were used a lactation rooms, dedicated lactation rooms are captioned “Lactation Room.” [file peerj-07-8168-s003.pdf]

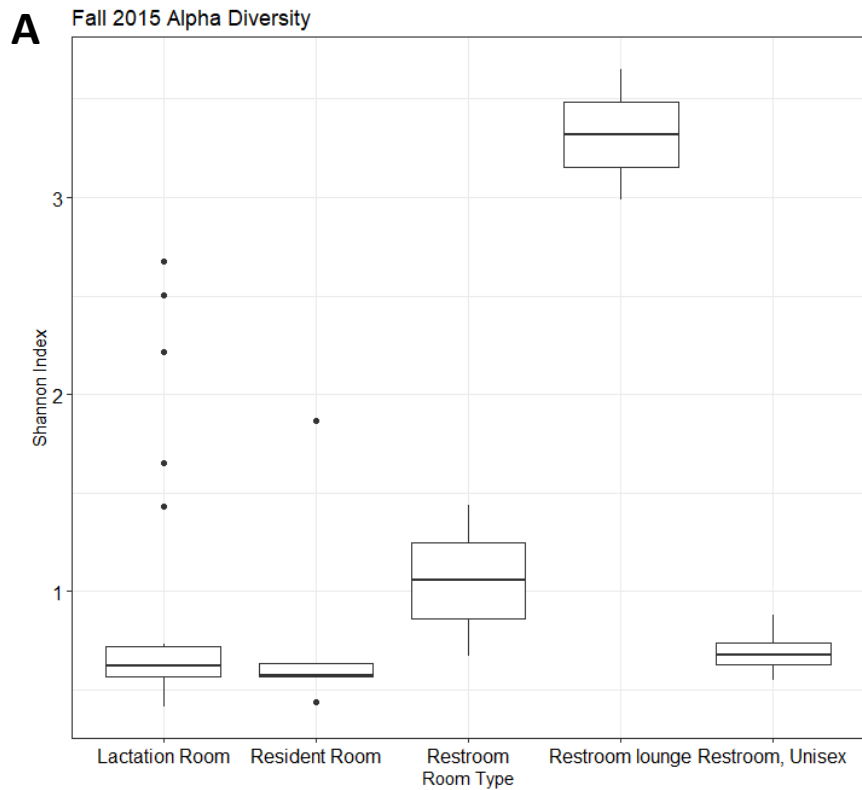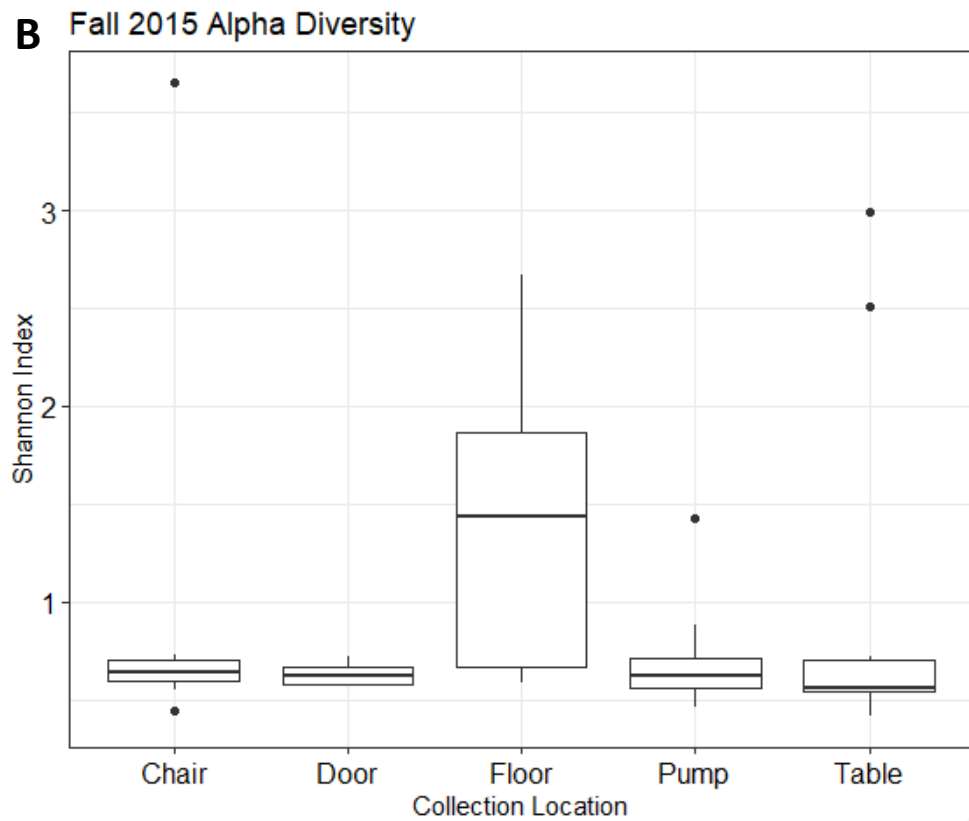

Supplement: Supplemental Information 4 — (A) Alpha diversity as measured by Shannon index by room type (p = 0.112). All rooms were used as lactation rooms, the label “Lactation Room” refers to dedicated lactation rooms. (B) Alpha diversity as measured by Shannon Index by sample collection location (p = 0.141). [file peerj-07-8168-s004.pdf]

# A

## Unweighted UniFrac Fall 2015 NMDS

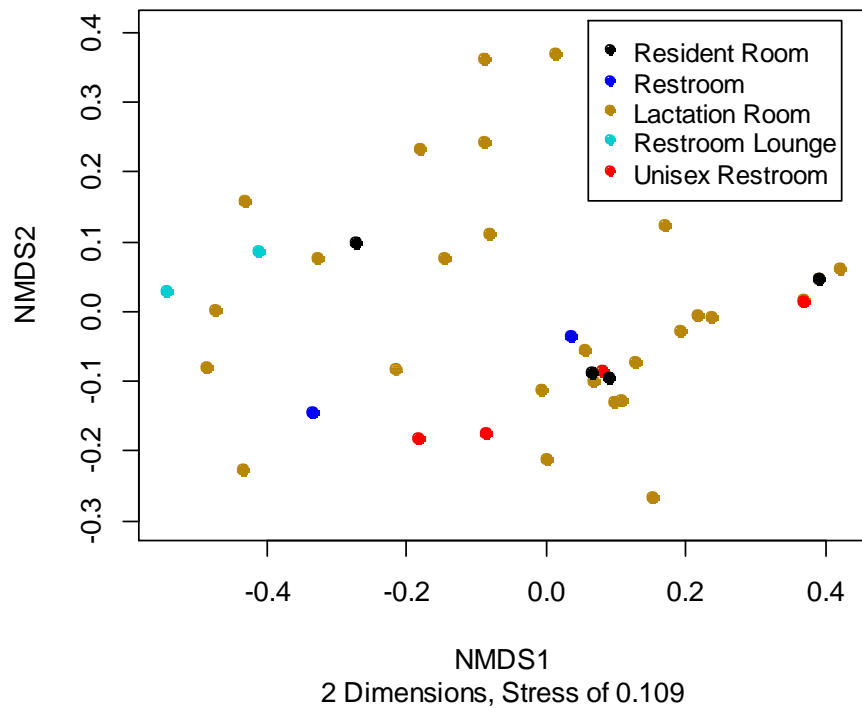

# B

## Unweighted UniFrac Fall 2015 NMDS

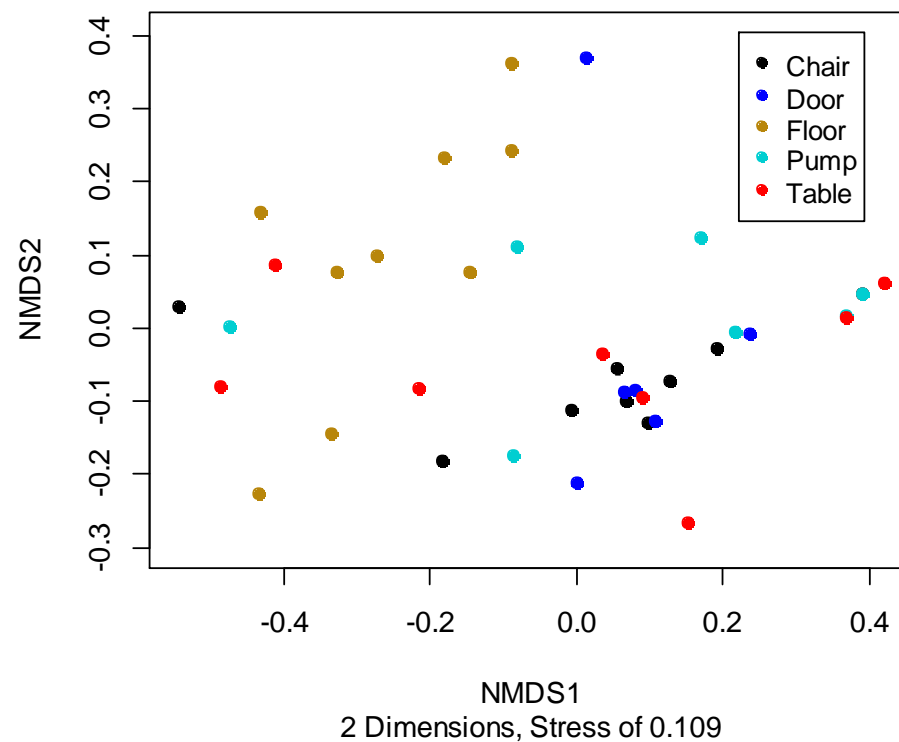

Supplement: Supplemental Information 5 — (A) Unweighted UniFrac NMDS with points colored by room type. All rooms were used as lactation rooms, the label “Lactation Room” refers to dedicated lactation rooms. (B) Unweighted UniFrac NMDS with points colored by sample collection location. [file peerj-07-8168-s005.pdf]

# A

## Weighted UniFrac Fall 2015 NMDS

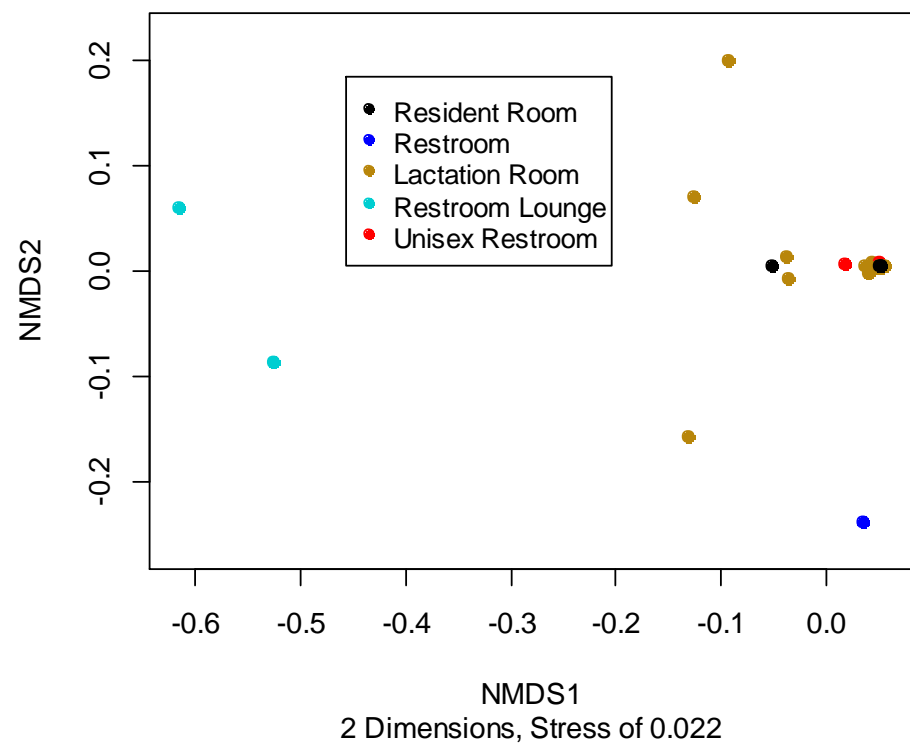

# B

## Weighted UniFrac Fall 2015 NMDS

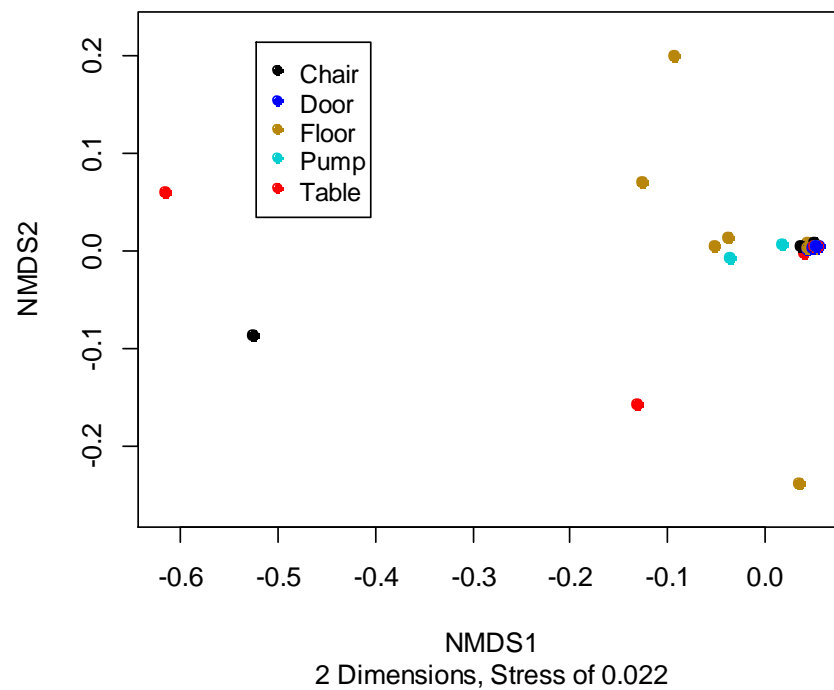

Supplement: Supplemental Information 6 — (A) Weighted UniFrac NMDS with points colored by room type. All rooms were used as lactation rooms, the label “Lactation Room” refers to dedicated lactation rooms. (B) Weighted UniFrac NMDS with points colored by sample collection location. [file peerj-07-8168-s006.pdf]

Winter 2016 Relative Abundance of Families by Room and Sample Location

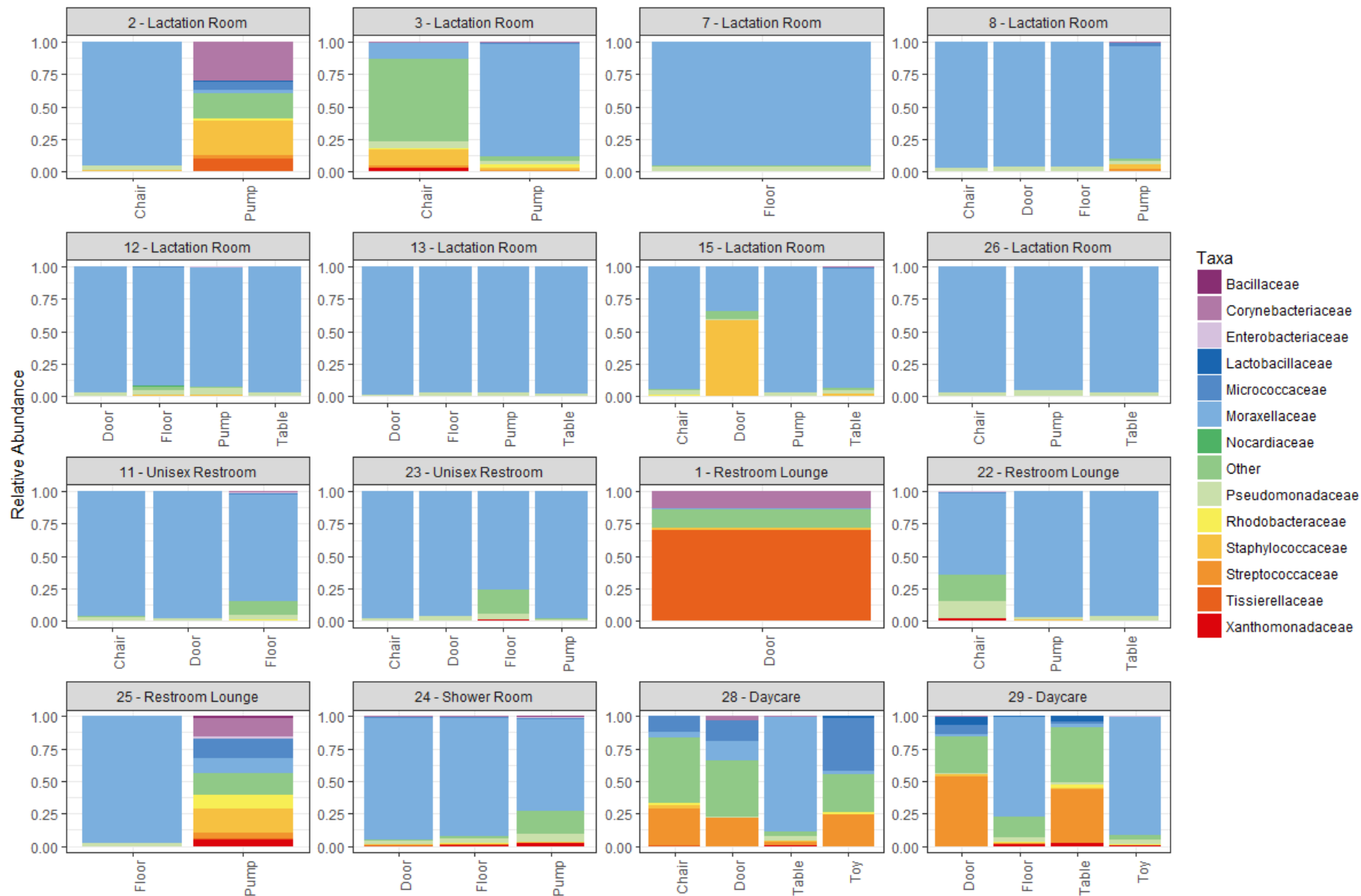

Supplement: Supplemental Information 7 — All rooms were daycares or used as lactation rooms, dedicated lactation rooms are captioned “Lactation Room.” [file peerj-07-8168-s007.pdf]

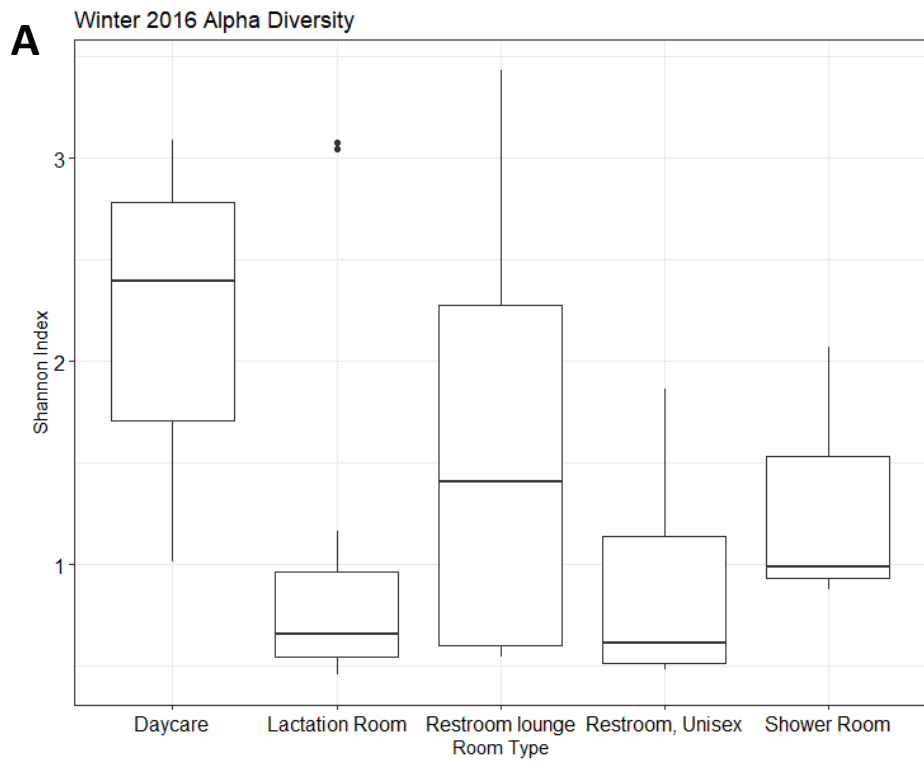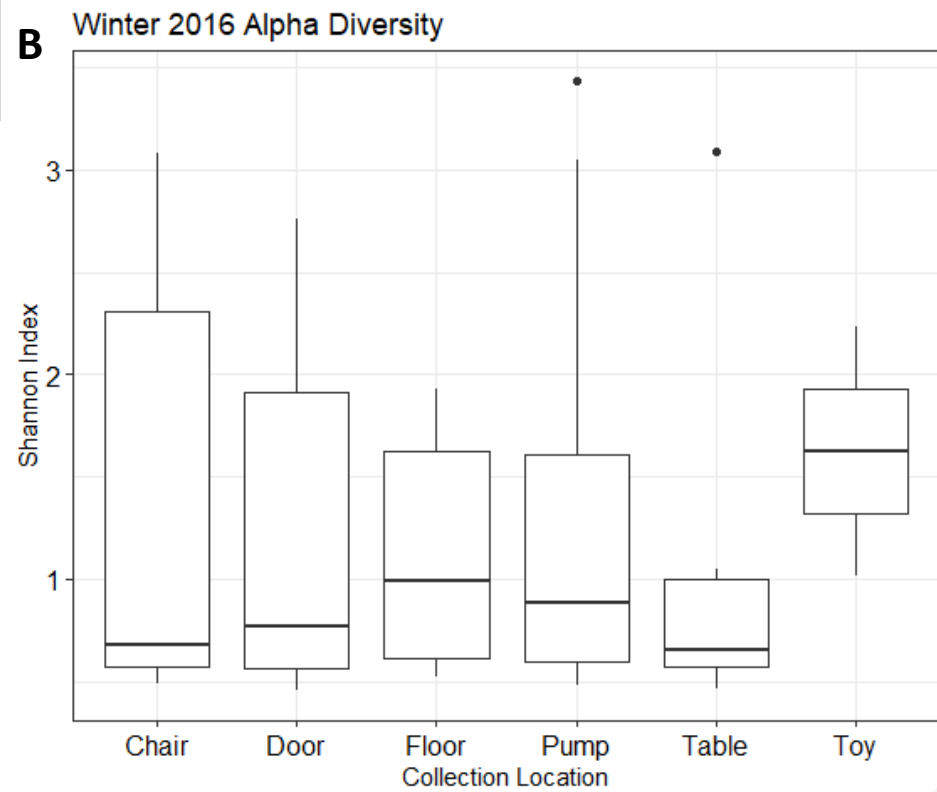

Supplement: Supplemental Information 8 — (A) Alpha diversity as measured by Shannon index by room type (Kruskal–Wallis, p = 0.0068, Dunn’s test indicated a difference was between daycares and lactation rooms (p = 0.0037) and between daycares and unisex restrooms (p = 0.013). All rooms were daycares or used as lactation rooms, the label “Lactation Room” refers to dedicated lactation rooms. (B) Alpha diversity as measured by Shannon Index by sample collection location (p = 0.90). [file peerj-07-8168-s008.pdf]

# A

## Unweighted UniFrac Winter 2016 NMDS

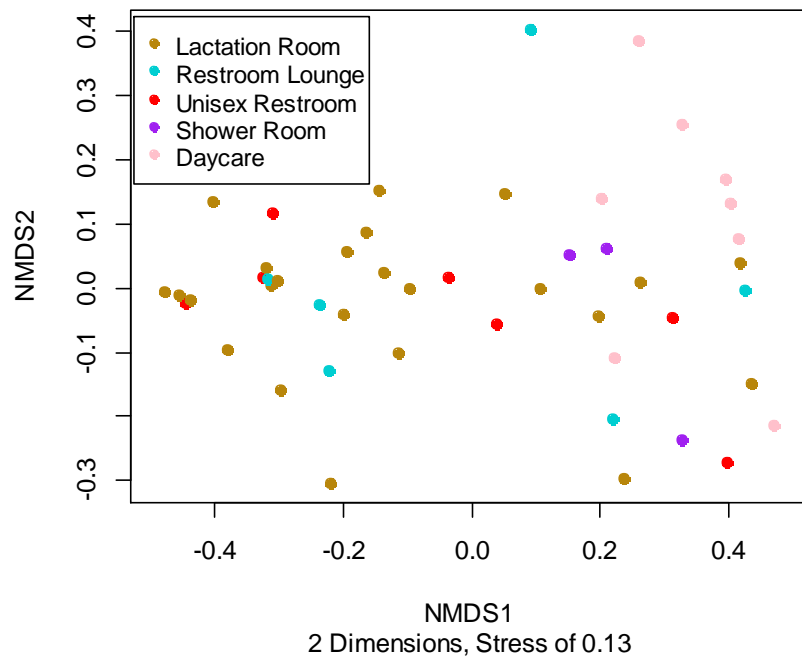

# B

## Unweighted UniFrac Winter 2016 NMDS

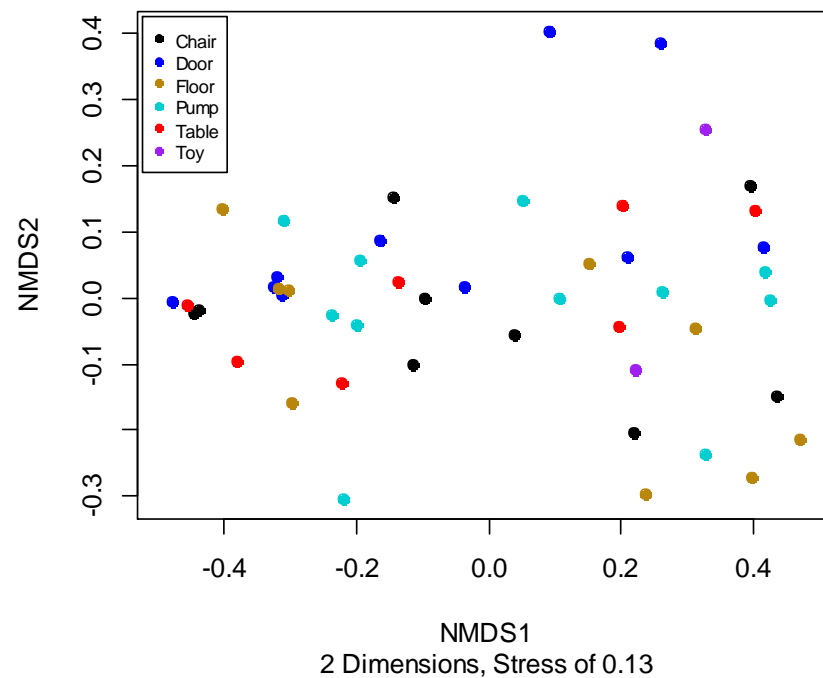

Supplement: Supplemental Information 9 — (A) Unweighted UniFrac NMDS with points colored by room type. All rooms were daycares or used as lactation rooms, the label “Lactation Room” refers to dedicated lactation rooms. (B) Unweighted UniFrac NMDS with points colored by sample collection location. [file peerj-07-8168-s009.pdf]

**A****Weighted UniFrac Winter 2016 NMDS**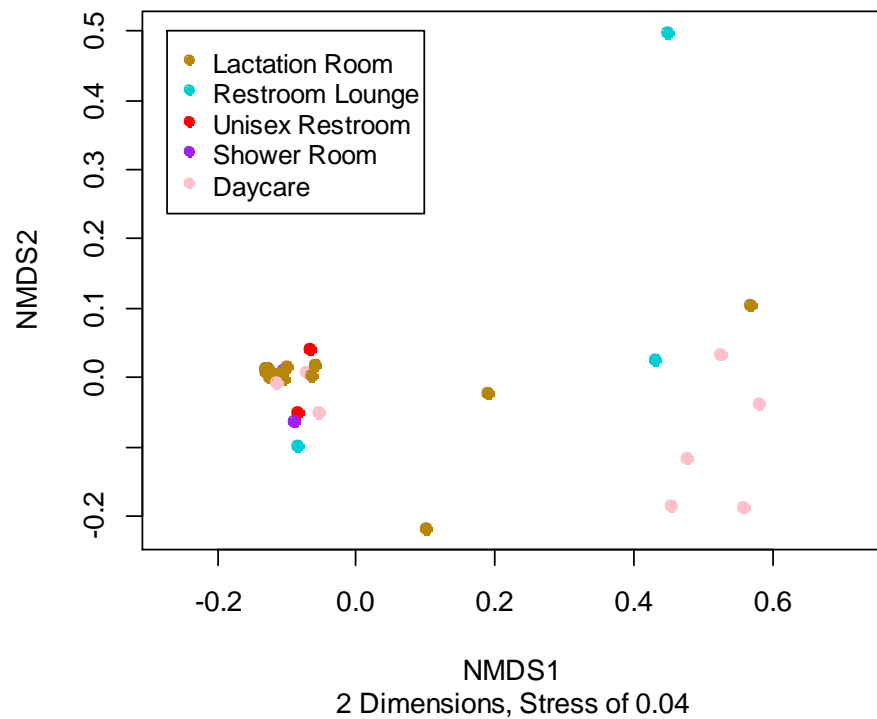**B****Weighted UniFrac Winter 2016 NMDS**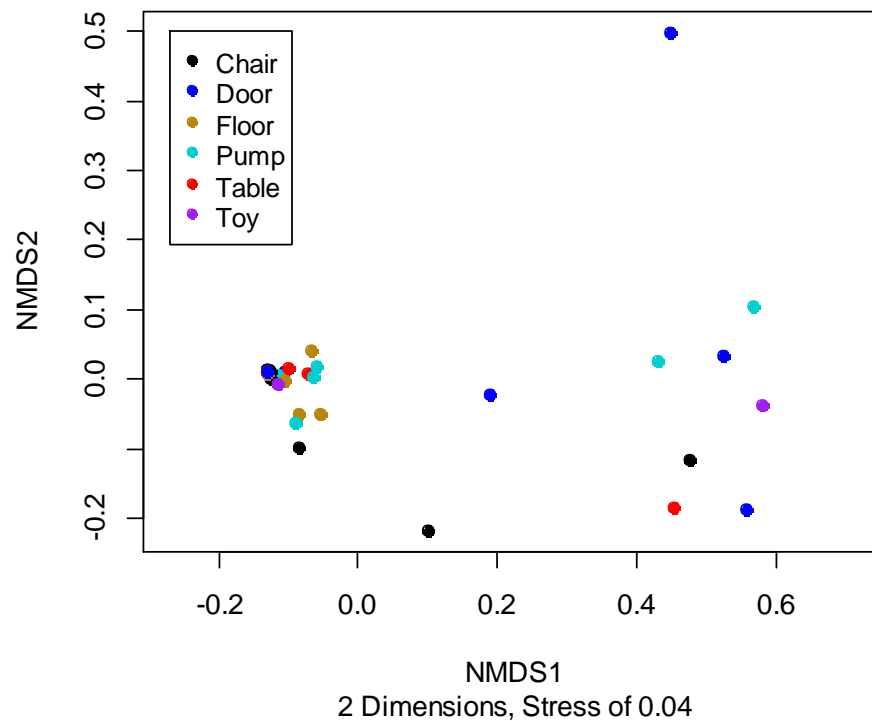

Supplement: Supplemental Information 10 — (A) Weighted UniFrac NMDS with points colored by room type. All rooms were daycares or used as lactation rooms, the label “Lactation Room” refers to dedicated lactation rooms. (B) Weighted UniFrac NMDS with points colored by sample collection location (PERMANOVA, p = 0.175). [file peerj-07-8168-s010.pdf]

Spring 2016 Relative Abundance of Families by Room and Sample Location

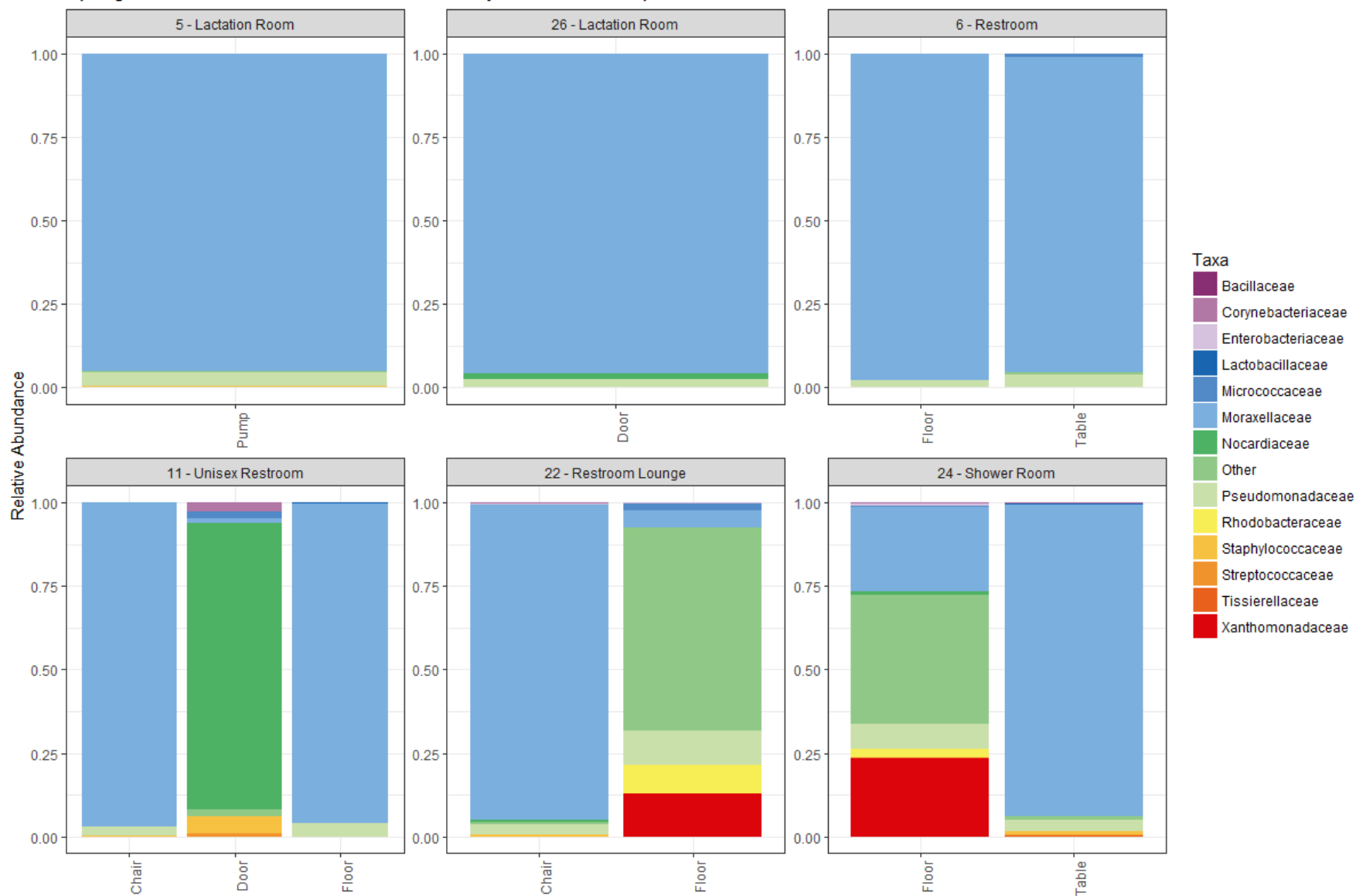

Supplement: Supplemental Information 11 — All rooms were used as lactation rooms, dedicated lactation rooms are captioned “Lactation Room.” [file peerj-07-8168-s011.pdf]

Fall 2016 Relative Abundance of Families by Room and Sample Location

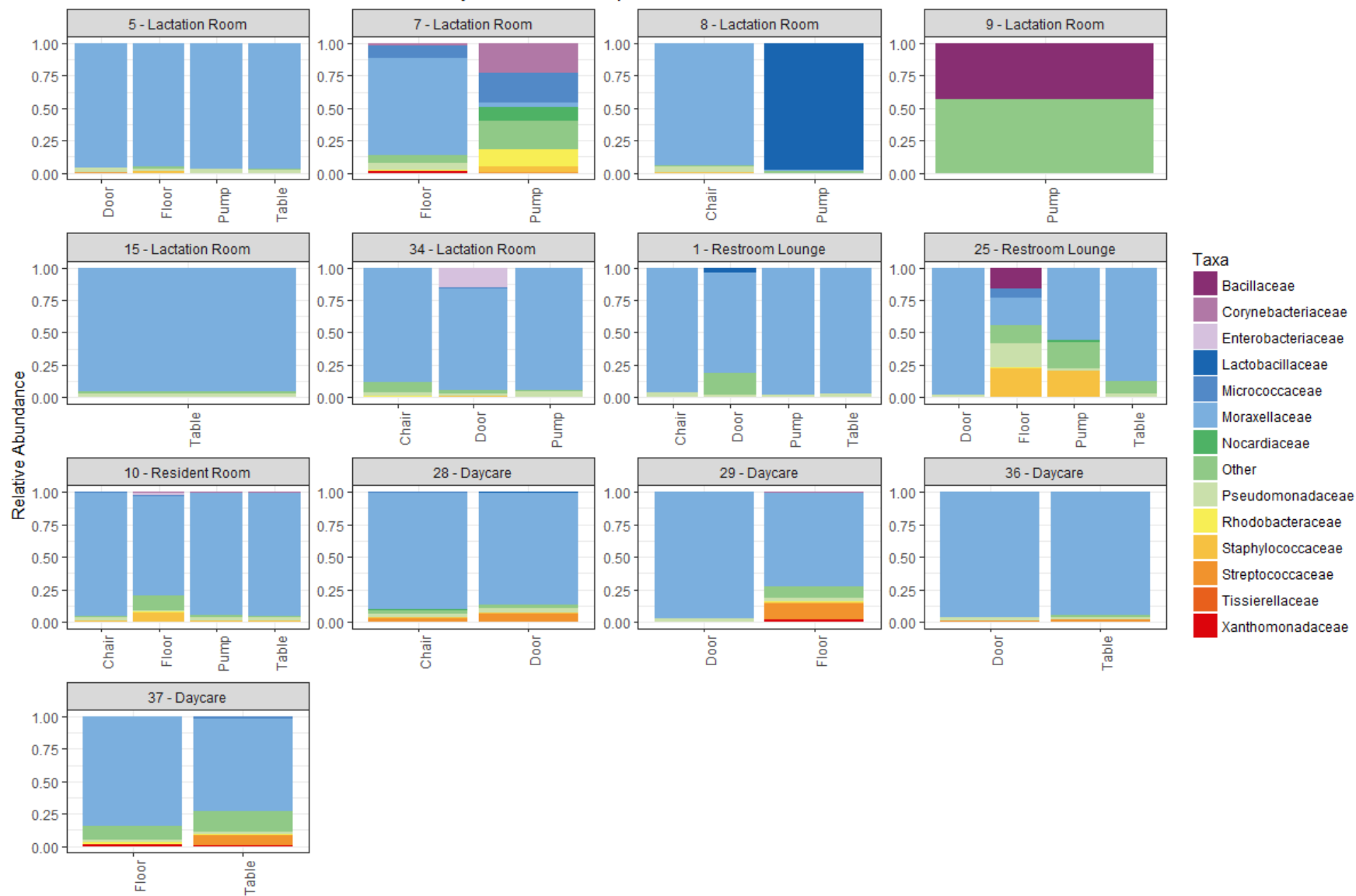

Supplement: Supplemental Information 12 — All rooms were daycares or used as lactation rooms, dedicated lactation rooms are captioned “Lactation Room.” [file peerj-07-8168-s012.pdf]

**A** Fall 2016 Alpha Diversity

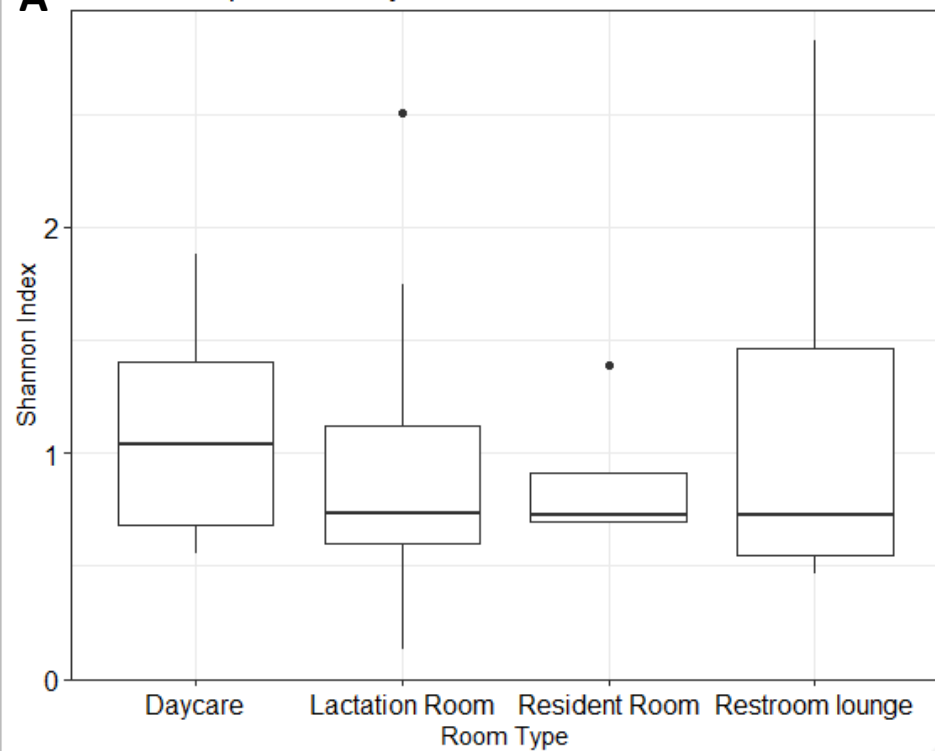

**B** Fall 2016 Alpha Diversity

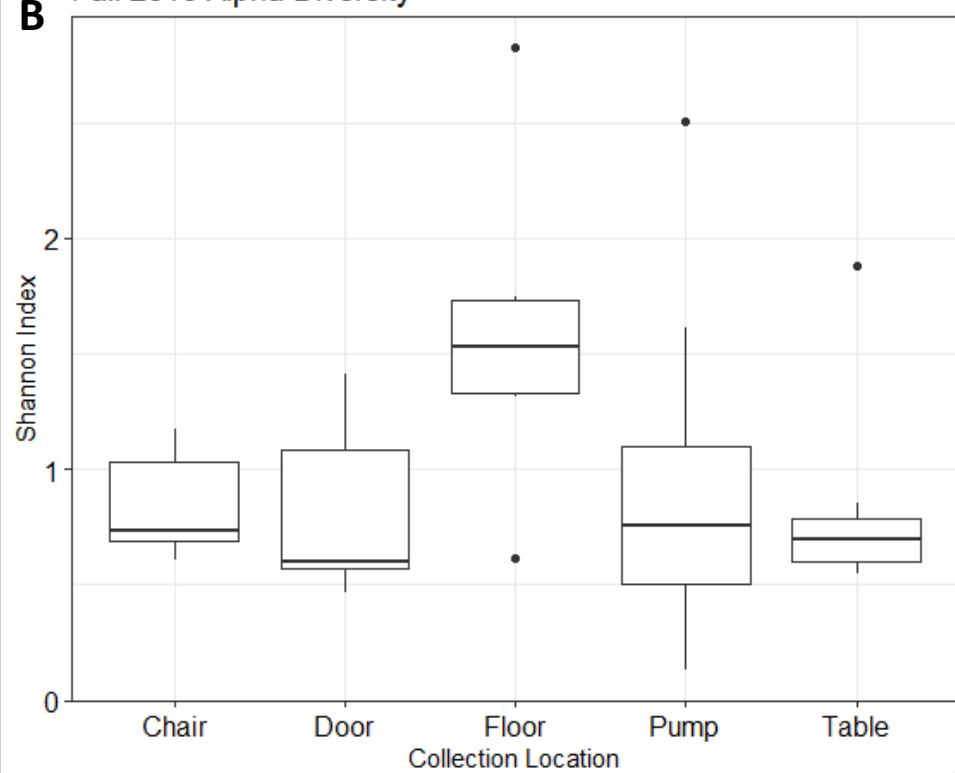

Supplement: Supplemental Information 13 — (A) Alpha diversity as measured by Shannon index by room type (p = 0.83). All rooms were daycares or used as lactation rooms, the label “Lactation Room” refers to dedicated lactation rooms. (B) Alpha diversity as measured by Shannon Index by sample collection location (p = 0.19). [file peerj-07-8168-s013.pdf]

**A****Unweighted UniFrac Fall 2016 NMDS**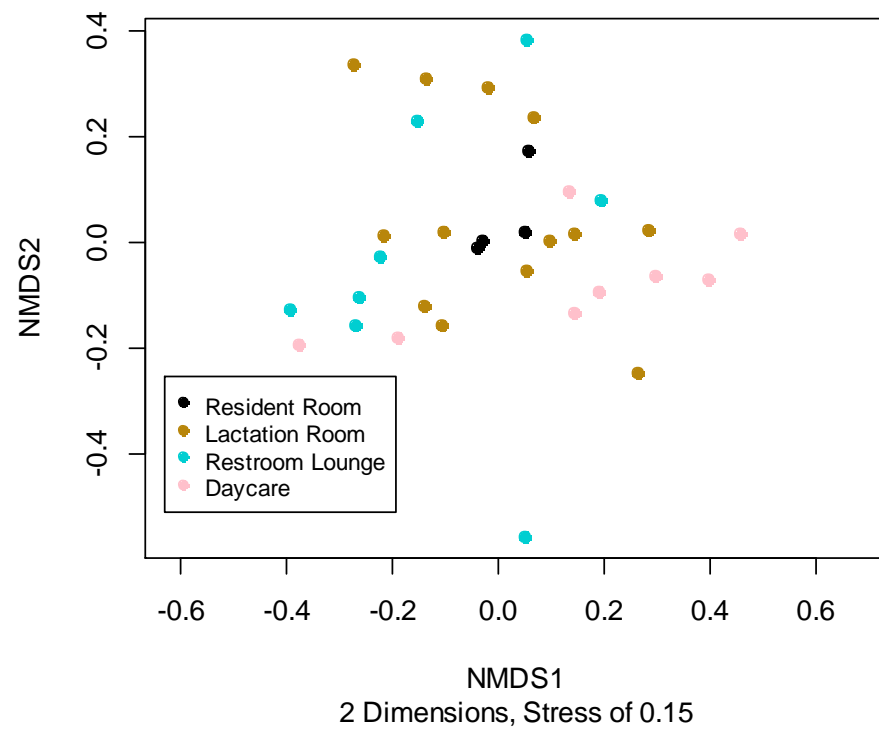**B****Unweighted UniFrac Fall 2016 NMDS**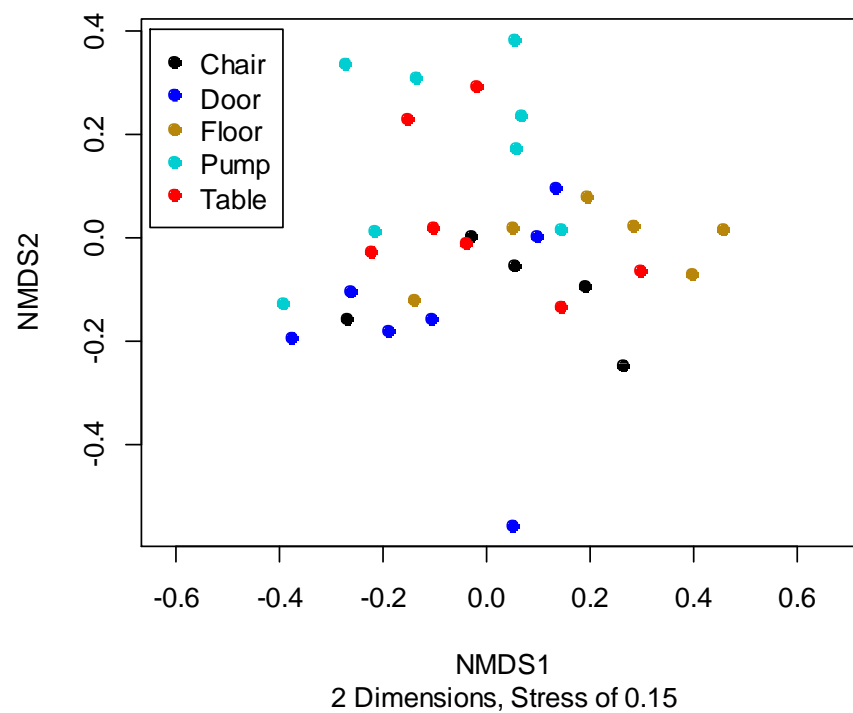

Supplement: Supplemental Information 14 — (A) Unweighted UniFrac NMDS with points colored by room type. All rooms were daycares or used as lactation rooms, the label “Lactation Room” refers to dedicated lactation rooms. (B) Unweighted UniFrac NMDS with points colored by sample collection location. [file peerj-07-8168-s014.pdf]

**A****Weighted UniFrac Fall 2016 NMDS**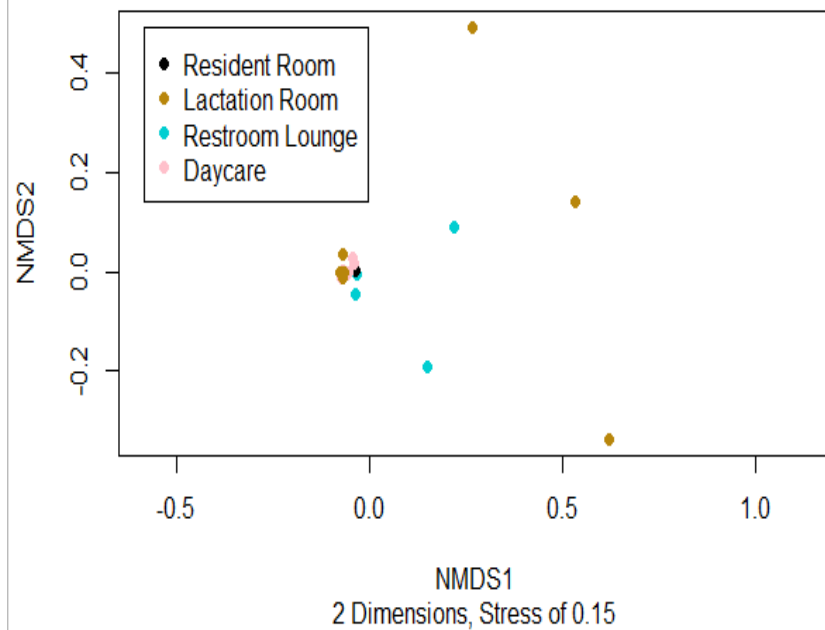**B****Weighted UniFrac Fall 2016 NMDS**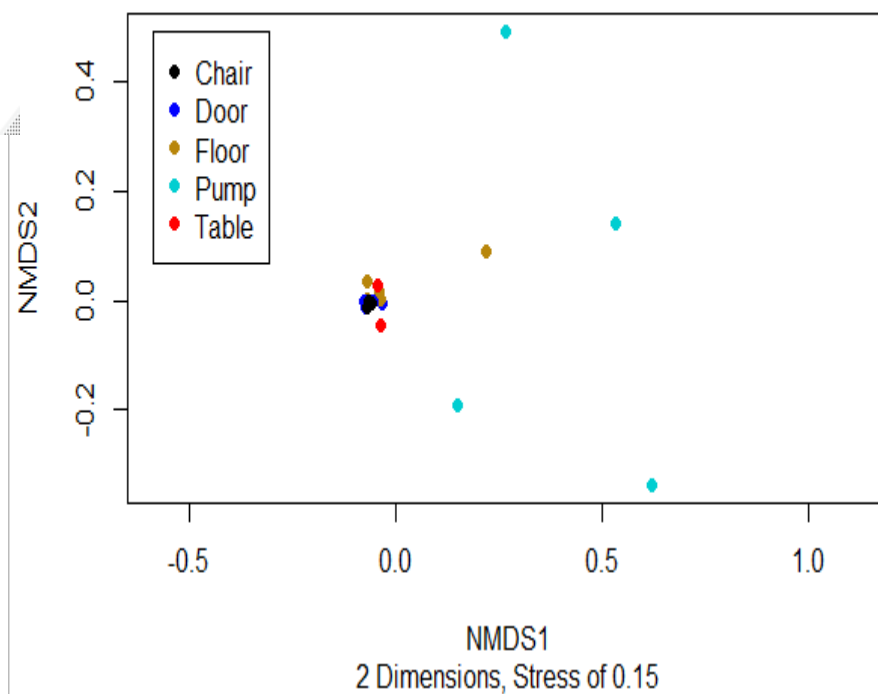

Supplement: Supplemental Information 15 — (A) Weighted UniFrac NMDS with points colored by room type. All rooms were daycares or used as lactation rooms, the label “Lactation Room” refers to dedicated lactation rooms. (B) Weighted UniFrac NMDS with points colored by sample collection location. Because of significant differences in betadispersion by sample collection location, sample collection location was not included in the PERMANOVA model. [file peerj-07-8168-s015.pdf]
